# Molecular docking analysis of bioactive molecules from herbs with snake venom phospholipase A2

**DOI:** 10.6026/973206300210290

**Published:** 2025-03-31

**Authors:** Karthik Nagarajan S., Siva Annamalai, Bharath Christian C.B.S, Rajamaheswari K., Lavanya A., Arunachalam K.

**Affiliations:** 1 Department of Clinical, National Institute of Siddha, Chennai, India; 2 Department of Clinical, Siddha Regional Research Institute, Puducherry, India; 3 Department of Clinical, Siddha Clinical Research Unit, Tirupati, India

**Keywords:** Molecular docking, network pharmacology, phospholipase A2

## Abstract

Snake venom, particularly phospholipase A2 (PLA2), exerts profound pathological effects, necessitating the development of potent therapeutic interventions. Therefore, it is of interest to the inhibitory potential of bioactive phytoconstituents from select medicinal herbs with PLA2. Analysis showed that Gymnemic acid, Aristolochic acid, Lupeol and Tocopherol are the best PLA2 inhibitors with strong binding and molecular interactions for further consideration.

## Background:

Snake venom comprises a diverse array of bioactive components, notably phospholipase A2 (PLA2), which contributes significantly to the toxicity observed in snakebites [1]. PLA2 enzymes can induce inflammatory responses, neurotoxicity and haemolytic activity, posing serious health risks to victims [2]. There exists a pressing need for effective antivenom treatments, especially in regions with a high prevalence of snakebite incidents. Recent investigations have turned towards herbal bioactive molecules as potential antidotes, leveraging their natural properties to mitigate venom effects [3]. Many herbal bioactive molecules in plants used in traditional medicines possess anti-inflammatory, antioxidant, analgesic and cytoprotective effects, making them suitable candidates for counteracting the detrimental impacts of PLA2 [4, 5, 6-7]. For instance, flavonoids and alkaloids derived from specific plants have demonstrated the ability to inhibit PLA2 activity, thereby reducing venom-induced damage [8]. In recent years, the quest for effective treatments against snake venom toxicity has shifted focus towards the potential of various herbal plants [9]. Among these, *Oxoxylum indicum (L.)* Benth. ex. Kurz *(Vaeliparutthi)*, *Aristolochia bracteolata* Lam. *(Aadutheenda Paalai)*, *Gymnema sylvestre* (Retz.) R. Br. ex Roem. & Schult. *(Sirukurinjan)*, *Boerhavia diffusa* L. *(Mookkirattai)* and *Corallocarpus epigaeus* (L.) S. C. Jain *(Aakaasakarudan Kizhangu)* have emerged as promising herbs, which have been mentioned in the Siddha textbook, *Nanju Murivu Nool* (the book that describes all types of poisoning treatment aspects, including plants, animals, metals and minerals), in the chapter of treatment for snake venom poisoning [10]. Research suggests that the anti-venom activity of these herbs could be attributed to their ability to inhibit key enzymatic functions, including the activity of phospholipase A2 (PLA2), a primary toxin in many snake venoms [11]. The mechanisms underlying these protective effects are complex and warrant further investigation, particularly through molecular docking studies to elucidate the interactions between bioactive compounds and venom enzymes [12]. Therefore, it is of interest to the inhibitory potential of bioactive phytoconstituents from select medicinal herbs with PLA2.

## Materials and Methods:

Docking analysis was performed using AutoDock version 4.2.6. In silico molecular docking was conducted to determine the binding energy between each ligand and the target protein Phospholipase A2 (PDB: 2QOG) for anti-venom therapy.

## Selection and preparation of ligands:

Eight bioactive compounds from the selected raw drugs of herbs such as scutellarein [13], gallic acid [14], piperonylic acid [15], aristolochic acid [16], gymnemic acid, lupeol [17], ascorbic acid and tocopherol [18] were collated from published research papers and a public database. Next, they were obtained in SDF format from https://pubchem.ncbi.nlm.nih.gov. By converting these ligands to PDB format using the OpenBabel program (http://openbabel.org/wiki/Main_Page), they are now ready for docking analysis. The torsion requirements needed for proper binding were then defined using the Autodock 4.2 6 application. Table 1 presents the vernacular and botanical names of the selected ligands for docking from the raw drugs of the herbs that were selected for docking with their PubChem ID. The ligands that were chosen for docking analysis are included in Table 2 along with their molar weight (g/mol), molecular formula, H bond donor, H bond acceptor and rotatable bonds.

## Protein preparation:

Figure 1(see PDF) shows the three-dimensional crystal structure of Phospholipase A2 (PDB: 2QOG). It was extracted from the Protein Data Bank (PDB), energy-minimized and converted to the appropriate PDBQT formats. The ligand molecules were first positioned, oriented and torsored randomly. When the device docked, all spinning torsions were relieved. Every docking experiment came from two distinct runs, each of which was intended to end after a maximum of 250000 energy assessments. The population size was set to 150. A translational step of 0.2 Å and quaternion and torsion steps of 5 were used in the search. Protein structures were cleaned by eliminating the lead components that were already there; water molecules were broken; polar hydrogens were included in the computation of Kollman's charges [19] and Auto Dock 4.2.6 software was used to describe the merging of non-polar and rotatable bonds. The selected ligands for molecular docking against Phospholipase A2 include scutellarein, piperonylic acid, aristolochic acid, gymnemic acid, lupeol, gallic acid, ascorbic acid and tocopherol. Their distinct 2D and 3D structures influence binding interactions with PLA2, as shown in Figure 2(see PDF).

## Molecular docking methodology:

Molecular docking was performed using AutoDock 4.2.6 after protein target preparation and ligand selection. The docking protocol involved grid box dimensions of 60x60x60 with a resolution of 0.375Å, flexible ligand conformations and selection of top-ranked binding poses based on binding energy and interaction profiles [20].

## Results and Discussion:

Table 3 summarizes the molecular docking studies of bioactive molecules against Phospholipase A2 (PDB: 2QOG), detailing the estimated free energy of binding, estimated inhibition constant (Ki), electrostatic energy, total intermolecular energy and interaction surface. Table 4 outlines the amino acid residue interactions of the bioactive molecules with phospholipase A2 [PDB: 2QOG].

Figure 3(see PDF) illustrates the docking poses of the ligands with the target receptor, phospholipase A2 (PDB: 2QOG). Figure 4(see PDF) presents the 2D interaction plot analysis of the ligands with the target receptor phospholipase A2 (PDB: 2QOG). Figure 5(see PDF) illustrates the hydrogen bond interactions along with core amino acid analysis of the ligands with the target receptor Phospholipase A2 (PDB: 2QOG). Based on the results of the computational analysis, it was concluded that the bioactive molecules like aristolochic acid, gymnemic acid, lupeol and tocopherol present in the herbs possess significant binding against the target enzyme phospholipase A2. Thereby, the selected bioactive molecules that inhibit the target enzyme Phospholipases A2 may occupy this active amino acid and could be able to block the hydrophobic channel, prevent the binding of the fatty acid necessary for the toxin allosteric activation during snake envenomation and act as a potential therapeutic agent for the management of snake bites. The present molecular docking study gives significant advancement in understanding the inhibitory potential of natural phytocomponents from traditional Siddha herbs against Phospholipase A2 (PLA2), a key enzyme involved in the toxicity caused by snake venom [21]. This enzyme is responsible for a range of deleterious effects, including myotoxicity, hemolysis and inflammation, which are characteristic of snake envenomation [1]. By targeting the core active sites of PLA2, the study aims to identify plant-derived compounds that could serve as effective therapeutic agents for snakebite management. Phospholipase A2 plays an important role in the degradation of phospholipids in cellular membranes, leading to the release of fatty acids and lysophospholipids, which are toxic to muscle tissues and other organs [22]. The enzyme's catalytic mechanism depends on crucial amino acid residues such as His48, Lys49, Tyr52 and Asp99 [23]. These residues are involved in stabilizing the enzyme-substrate complex, allowing the toxin to exhibit its detrimental effects. In this study, the in silico docking analysis highlighted the binding interactions of eight bioactive compounds, with particular emphasis on aristolochic acid, gymnemic acid, lupeol and tocopherol. These compounds exhibited the most potent binding affinities and significant hydrogen bonding with the aforementioned critical residues of PLA2, which implies that they could effectively hinder the enzyme's toxic function.

Aristolochic acid displayed a notable binding affinity of -7.29 kcal/mol with an inhibition constant (Ki) of 4.50 µM. The docking analysis revealed its ability to form two key interactions with the active site residues. Aristolochic acid is known for its anti-inflammatory properties, which could be synergistic in counteracting venom-induced edema and inflammation [24]. The strong binding interaction suggests that aristolochic acid could disrupt the structural integrity of PLA2, preventing it from catalyzing its toxic substrates. In all the compounds tested, gymnemic acid demonstrated the highest binding affinity (-12.33 kcal/mol) and the lowest inhibition constant (923.65 pM). Its molecular structure allows for multiple hydrogen bonds with the active site, particularly with residues such as leucine, tryptophan and aspartate. The ability of gymnemic acid to occupy key binding sites could effectively block the hydrophobic channel of PLA2, thus preventing the enzyme from accessing its fatty acid substrates. This compound may be a lead one for further drug development. Lupeol is a well-known bioactive compound with various pharmacological activities, including anti-inflammatory and antioxidant effects [25-26]. In this study, Lupeol showed a binding affinity of -7.11 kcal/mol and interacted with two active site residues. Its ability to form stable interactions with PLA2 suggests that it could inhibit the enzyme's function, reducing the local tissue damage and inflammation caused by snake venom.

As an antioxidant, tocopherol plays a crucial role in neutralizing free radicals generated during venom-induced oxidative stress [27]. In the docking study, tocopherol exhibited a binding energy of -6.30 kcal/mol and formed interactions with two of the core amino acid residues. Its role in scavenging reactive oxygen species (ROS) could further enhance its therapeutic potential by protecting cells from venom-induced oxidative damage. Therefore, the results of this current study suggest that these phytocomponents can effectively interact with the catalytic domain of PLA2, particularly by forming hydrogen bonds with key amino acid residues. This binding could result in the occupation of the enzyme's hydrophobic channel, thereby preventing the entry of fatty acids that are necessary for the allosteric activation of PLA2. By blocking this hydrophobic channel, the phytocomponents could inhibit the enzyme's function, reducing the severity of envenomation symptoms such as myonecrosis, edema and inflammation. Additionally, some of these compounds have anti-inflammatory and antioxidant activities, which may offer dual functionality by not only inhibiting PLA2 but also mitigating oxidative stress. The antioxidant properties of these compounds could play a crucial role in reducing secondary effects of snake venom like systemic inflammation and cellular damage. Conventional treatments for snakebite envenomation primarily involve the administration of antivenom derived from equine or ovine sources. Despite their effectiveness, conventional treatments face several limitations, like access can be limited in rural or underserved areas, they are very expensive and some patients experience adverse reactions to antivenoms and antivenoms may not effectively neutralize all types of venom due to species specificity [28]. The plant-based nature of these compounds presents an opportunity to develop cost-effective, easily accessible anti-venom therapies, particularly in regions where snakebite incidence is high and conventional anti-venoms are either unavailable or ineffective against certain snake species. Plant-derived inhibitors like those identified in this study could offer a sustainable alternative, especially in rural and resource-limited settings. The Siddha system of medicine has plenty of traditional formulations to combat snakebite; most of them are prepared by the herbals, particularly *Oxoxylum indicum (L.)* Benth. Ex. Kurz *(Vaeliparutthi)*, *Aristolochia bracteolata* Lam. *(Aadutheenda Paalai)*, *Gymnema sylvestre* (Retz.) R. Br. ex Roem. and Schult. *(Sirukurinjan)*, *Boerhavia diffusa* L *(Mookkirattai)* and *Corallocarpus epigaeus* (L) S.C. Jain *(Aakaasakarudan Kizhangu)*. Through this current in silico approach, these herbs are proven as potent herbs against snakebite envenomations. Overall, the docking results substantiate the hypothesis that herb.al bioactive molecules can serve as potential inhibitors of PLA2, which is crucial in delineating the pathway for the mitigation of snake venom toxicity. The study emphasizes not only the traditional medicinal value of these herbs but also provides a scientific basis for their efficacy, illustrating how computational methods can be employed to validate ethnobotanical knowledge.

## Limitations and future directions:

The actual efficacy of these phytocomponents must be validated through *in vitro* and *in vivo* studies to assess their pharmacokinetics, bioavailability and safety profiles. The specific mechanisms by which these compounds inhibit PLA2 in a physiological environment are needed to be further explored. The potential synergy between these compounds and existing anti-venoms could be investigated, as combination therapies might enhance therapeutic outcomes and reduce the dosage of conventional anti-venom required, thereby minimizing side effects.

## Conclusion:

Aristolochic acid, gymnemic acid, lupeol and tocopherol exhibit strong inhibitory potential against phospholipase A2, making them promising candidate for the development of new anti-venom therapies. These phytocomponents could mitigate the toxic effects of snake venom, offering a novel, plant-based approach to snakebite management by blocking the enzymatic activity of PLA2. However, experimental validation and clinical trials are needed to fully harnessing the therapeutic potential of these natural compounds.

## Sponsorship and financial support:

None

## Figures and Tables

**Table 1:** Selected ligands for docking from the raw drugs of the selected herbs

**Vernacular Name (Tamil)**	**Botanical name of the herbs**	**Selected Phytochemicals [13-18]**	**PUBCHEM ID**
*Vaelipparutthi*	*Oxoxylum indicum* (L.) Benth. ex. Kurz	Scutellarein	5281697
*Mookkirattai*	*Boerhavia diffusa* L.	Gallic Acid	370
*Aadutheenda Paalai*	*Aristolochia bracteolata* Lam.	Piperonylic acid	7196
		Aristolochic acid	2236
*SiruKurinjhan*	*Gymnema sylvestre* (Retz.) R. Br. ex Roem. & Schult.	Gymnemic acid	11953919
		Lupeol	259846
*Aakaasa Karudan*	*Corallocarpus epigaeus* (L.) S. C. Jain	Ascorbic acid	54670067
*Kizhangu*		Tocopherol	-

**Table 2:** Properties of ligands selected for docking analysis

**Compound**	**Molar weight (g/mol)**	**Molecular Formula**	**H Bond Donor**	**H Bond Acceptor**	**Rotatable bonds**
Scutellarein	462.4	C_21_H_18_O_12_	7	12	4
Piperonylic acid	166.13	C_8_H_6_O_4_	1	4	1
Aristolochic acid	341.27	C_17_H_11_NO_7_	1	7	2
Gymnemic acid	807	C_43_H_66_O_14_	7	14	10
Lupeol	426.7	C_30_H_50_O	1	1	1
Gallic Acid	170.12	C_7_H_6_O_5_	4	5	1
Ascorbic acid	176.12	C_6_H_8_O_6_	4	6	2
Tocopherol	472.7	C_31_H_52_O_3_	0	3	14

**Table 3:** Summary of the molecular docking studies of the selected bioactive molecules against Phospholipases A2 [PDB: 2QOG]

**Compounds**	**Est. Free Energy of Binding (kcal/mol)**	**Est. Inhibition Constant, Ki**	**Electrostatic Energy (kcal/mol)**	**Total Intermolecular Energy (kcal/mol)**	**Interaction Surface**
Gymnemicc acid	-12.33	923.65 pM	-0.15	-9.89	777.91
Scutellarein	-8.67	440.62 nM	-1.26	-7.77	596.434
Aristolochic acid	-7.29	4.50 uM	-1.18	-8.28	554.1
Lupeol	-7.11	6.18 uM	-0.05	-7.79	622.792
Tocopherol	-6.3	23.91 uM	-0.02	-7.35	580.841
Gallic Acid	-5.33	124.31 uM	-1.08	-4.87	342.747
Ascorbic acid	-5.59	79.52 uM	-0.21	-4.52	417.854
Piperonylic acid	-4.79	306.93 uM	-0.23	-5.09	422.126

**Table 4:** Amino acid residue interaction of lead phospholipase A2 [PDB: 2QOG] with selected bioactive molecules

**Compounds**	**Inter actions**	**Amino acid Interactions**								
Scutellarein	1	1 SER	02 LEU	31 TRP	52 TYR	67 ASN	70 TRP			
Piperonylic acid	0	05 PHE	06 ASN	09 ILE	18 ALA	22 TYR	23 ALA	31 TRP	45 CYS	
Aristolochic acid	2	01 SER	02 LEU	31 TRP	49 ASN	52 TYR	67 ASN	70 TRP		
Gymnemic acid	2	02 LEU	31 TRP	49 ASN	52 TYR	67 ASN	70 TRP			
Lupeol	2	02 LEU	31 TRP	49 ASN	52 TYR	67 ASN	70 TRP			
Gallic Acid	1	52 TYR	67 ASN	70 TRP						
Ascorbic acid	0	02 LEU	05 PHE	06 ASN	09 ILE	22 TYR	23 ALA	29 CYS	31 TRP	45CYS
Tocopherol	2	02 LEU	31 TRP	49 ASN	52 TYR	67 ASN	70 TRP			
